# Co-Production of Cellulose Nanocrystals and Fermentable Sugars Assisted by Endoglucanase Treatment of Wood Pulp

**DOI:** 10.3390/ma11091645

**Published:** 2018-09-07

**Authors:** Jing Dai, Michael Chae, Dawit Beyene, Christophe Danumah, Frank Tosto, David C. Bressler

**Affiliations:** 1 Department of Agricultural, Food and Nutritional Science, University of Alberta, Edmonton, AB T6G 2P5, Canada; janetdai100@outlook.com (J.D.); mchae@ualberta.ca (M.C.); dbeyene@ualberta.ca (D.B.); 2 Biomass Conversion and Processing Technologies, InnoTech Alberta, Edmonton, AB T6N 1E4, Canada; christophe.danumah@innotechalberta.ca (C.D.); frank.tosto@innotechalberta.ca (F.T.)

**Keywords:** cellulose nanocrystals, endoglucanase treatment, acid hydrolysis, wood pulp, degree of crystallinity, fermentable sugars

## Abstract

In this study, fermentable sugars and cellulose nanocrystals (CNCs) were co-produced from endoglucanase treatment of wood pulp, followed by acid hydrolysis. Enzymatic hydrolysis was performed using two endoglucanases differentiated by the presence or absence of a cellulose-binding domain (CBD). The enzyme with an intact CBD gave the higher glucan conversion (up to 14.1 ± 1.2 wt %) and improved the degree of crystallinity of the recovered wood pulp fiber (up to 83.0 ± 1.0%). Thus, this endoglucanase-assisted treatment successfully removed amorphous content from the original cellulosic feedstock. CNC recovery (16.9 ± 0.7 wt %) from the feedstock going into the acid hydrolysis was improved relative to untreated pulp (13.2 ± 0.6 wt %). The mass loss from enzymatic treatment did not cause a decrease in the CNC yield from the starting material. The characteristics of CNCs obtained through acid hydrolysis (with or without enzyme treatment of pulp) were analyzed using X-ray diffraction, transmission electron microscopy, dynamic light scattering, Fourier transform infrared spectroscopy, and differential scanning calorimetry as characterization techniques. The CNCs generated through acid hydrolysis of endoglucanase-treated wood pulp displayed comparable properties relative to those generated using untreated pulp. Thus, endoglucanase treatment can enable co-production of CNCs and sugars for biofuel fermentation.

## 1. Introduction

Over the last decade, the potential for the development of nanomaterials from the forest sector has become apparent and achievable. Additionally, the market demand for some traditional forest products, such as pulp and paper, has greatly declined [1]. The production of high-performance nanomaterials provides opportunities for the development of more valuable forestry products to sustain the forest-based economy [2]. Cellulose nanocrystals (CNCs) are nanomaterials that are derived from the most abundant renewable polymer resource—cellulose [3]. Cellulose can be isolated from wood by a kraft pulping process [4]. In its natural form, cellulose is primarily comprised of two main forms: loosely-packed (amorphous) cellulose fibers and highly structured (crystalline) cellulose fibers. CNCs are formed from the elementary building blocks of highly ordered crystalline cellulose with lengths and widths in the nanoscale range [5]. Particles and matter at these dimensions display unique and novel properties that are unlike those displayed by particles at the larger dimensions of their precursors [2]. CNCs have very high tensile strength, axial Young’s modulus, and are potentially stronger than Kevlar [3]. They also have low density, a high aspect ratio, are biodegradable, and can be modified through reactive –OH groups found onto their surfaces [6,7]. All these properties make CNCs a good reinforcement in nanocomposites to improve mechanical and barrier properties, and several studies have been written in this area [8,9,10,11]. Recently, increasing interest has been focused on fully bio-based, biodegradable, and robust polymeric systems. This may be achieved by introducing CNCs into natural or bio-based plastics, the latter of which can be produced from starch, polylactic acid, or polyhydroxyalkanoates [12].

Among the several methods for isolating CNCs, acid hydrolysis is the most well-known and widely used [9,13,14]. This process breaks the disordered amorphous regions of cellulose, releasing well-defined crystals, and is based on the faster hydrolysis kinetics presented by the amorphous regions as compared to the crystalline ones [15]. Other methods for CNC production, such as high-pressure homogenization, microwave or ultrasonic treatment, and ionic liquid extraction, have also been studied [16,17,18,19], but the associated high energy and production costs have to be considered when these methods are applied. The use of enzymatic hydrolysis for CNC production is rather limited, with most of these studies focused on preparation of microfibrillated cellulose (MFC) [20,21]. However, the use of enzyme treatment in combination with mechanical processing for CNCs production has been widely studied [10,22,23]. Usually, MFC and CNCs coexist in the suspension, with their proportions, as well as the aspect ratio of resulting CNCs, being dependent on the enzyme treatment condition [3,22].

The generic term cellulase refers generally to a system of three classes of enzymes: endoglucanase, exoglucanase (most commonly referred to as cellobiohydrolase), and β-glucosidase. The combined actions of these enzymes causes the efficient degradation of cellulose [24]. In the literature, endoglucanases are described as enzymes that randomly attack the internal glycosidic bonds within an unbroken glucan chain in the amorphous regions; the reducing and non-reducing chain ends then become the substrate for cellobiohydrolase (CBHI/Cel7A and CBHII/Cel6A, respectively), which release cellobiose units. β-Glucosidase hydrolyzes the cellobiose into glucose as an end product [25,26,27]. The cellulase structure has two main components: the catalytic domain and the cellulose-binding domain (CBD). The catalytic domain is the active site for hydrolytic cleavage of glycosidic bonds, while the CBD facilitates the binding and concentration of enzyme on the cellulose for effective activity [28,29]. Previous studies have determined that the CBD was capable of binding to other carbohydrates and, thus, this domain is also referred to as a carbohydrate-binding module (CBM) [30]. Cel12A family is the only cellulase that is naturally lacking CBD, with no known function [31].

Endoglucanases have been shown to have relatively mild activity, which is suitable for hydrolysis of amorphous regions of cellulose without complete hydrolysis to glucose, while preserving the crystalline cellulose domain [21,22]. In a previous study from our group, we reported higher CNC yields and less acid consumption using cellulase-treated cellulose as crystalline-enriched feedstock for acid hydrolysis [32]. Furthermore, this enzyme treatment resulted in a sugar stream that could be fermented to biofuels such as ethanol. However, these experiments made use of highly processive cellulase cocktails that have been designed to maximize hydrolysis of cellulosic materials, including the more recalcitrant crystalline regions. Consequently, some of the crystalline precursors of CNCs may have been degraded, ultimately leading to lower CNC yields. To avoid degradation of such crystalline regions, we examined the use of endoglucanases that specifically target amorphous regions of cellulose.

This work is aimed at evaluating endoglucanase hydrolysis of wood pulp and determining the effects of endoglucanase use on co-production of fermentable sugars, as well as CNCs, through subsequent acid hydrolysis. We theorize that the degradation of amorphous cellulose by endoglucanase hydrolysis would increase the concentration of the crystalline cellulosic precursors for CNC isolation relative to the starting material, leading to more efficient acid hydrolysis.

## 2. Materials and Methods

### 2.1. Feedstock

Northern bleached hardwood kraft (NBHK) pulp was oven-dried and cut into square pieces (roughly 8 × 6 × 1 mm, in length, width and thickness) by a pulp chopper (Cutting Machine G45L1, Pierret, Corbion, Belgium). The pulp, which was kindly provided by Alberta Pacific Forest Industries Inc. or Al-Pac (Edmonton, AB, Canada), had a moisture content of 5.9 ± 0.1%. A two-step acid digestion procedure was carried out in quadruplicate, according to Jain et al., for pulp compositional analysis [33]. NBHK pulp had 78.0 ± 0.8% glucan, 15.7 ± 1.0% xylan, 0.3 ± 0.1% extractives, and 0.8 ± 0.2% acid soluble lignin. It should be noted that routine compositional analysis following the standard TAPPI protocol (T 203) based on a two-step alkaline extraction, as determined by InnoTech Alberta’s Biomass Conversion and Processing Technologies Unit for quality control purposes, gives 92.5% alpha cellulose, 1.8% acid insoluble lignin, 0.6% ash, and 0.4% extractives.

### 2.2. Enzymes and Specific Activity

Enzymatic hydrolysis was performed using two proprietary, non-commercially purified endoglucanase preparations: an endo-1,4-beta-glucanase (Endo-CBD+: NS 51137), and a mutant form of the same enzyme in which the cellulose-binding domain had been removed (Endo-CBD−: NS 51172). These enzymes were graciously provided by Novozymes^®^ A/S (Bagsvaerd, Denmark). The optimum pH of the enzyme treatment systems employed for Endo-CBD+ and Endo-CBD− were established to be 7.0 and 4.0, respectively. Endoglucanase activities were determined following the carboxymethyl cellulose (CMC) assay [34], which facilitated equalization of Endo-CDB+ and Endo-CDB− loading. Based on the results from the CMC assay, a range of enzyme loadings (0.2, 0.4, 0.6, 0.8, 1.0, and 5.0 U/g of pulp) were used for the experiments described below.

Since endoglucanases release long chain polysaccharides rather than mono- or disaccharides, total sugar release was measured following complete hydrolysis of sugars in the liquid phase recovered after endoglucanase treatment. This was achieved using a highly-processive proprietary cellulase cocktail solution (NS 51129) provided by Novozymes^®^ A/S (Bagsvaerd, Denmark), which was the same enzyme employed by Beyene et al. [32].

### 2.3. Enzymatic Hydrolysis

A citrate-phosphate buffer was used for enzyme hydrolysis of the wood pulp. The pH of the buffer was adjusted to 7.0 for Endo-CBD+ and 4.0 for Endo-CBD− by formulating different proportions of stock solutions of 0.1 M citric acid (99.5%; Sigma Aldrich, St. Louis, MO, USA) and 0.2 M dibasic sodium phosphate (99%; Fisher Scientific, Hampton, NH, USA).

Wood pulp (10 g, dry weight) was hydrolyzed with enzymes in citrate-phosphate buffer (100 mL) at 50 °C for 72 h in a shaking (100 rpm) water bath. The reaction was terminated by heating in boiling water for 10 min. Pulp samples that were suspended in buffer without addition of enzymes and treated under the same condition served as the negative control. All experiments were done in triplicates.

Following endoglucanase or mock treatment, the resulting slurries were centrifuged at 33,700× *g* for 10 min. The solid fraction was recovered and subsequently subjected to standard acid hydrolysis for production of CNCs (as described in Section 2.5). An aliquot of the liquid fraction (5 mL), recovered as enzyme hydrolysate, was subjected to an additional step of enzymatic hydrolysis using NS 51129 to promote complete saccharification of the released long and/or short cellulose chains. This subsequent enzymatic hydrolysis was conducted at 50 °C for 72 h in a shaking water bath (100 rpm). The measured sugar concentrations were used to calculate the recoverable sugar yield from the endoglucanase treatment.

### 2.4. High Performance Liquid Chromatography (HPLC)

Monosaccharides were quantified using an HPLC system (HPLC, 1200 series, Agilent Technologies, Mississauga, ON, Canada) equipped with a refractive index detector (1100 series, Agilent Technologies, Mississauga, ON, Canada) and an Aminex HPX-87P column (Bio-Rad, Hercules, CA, USA). The analyses were carried out using HPLC water (Sigma Aldrich, St. Louis, MO, USA) as the mobile phase at a temperature of 65 °C and a flow rate of 0.5 mL/min for 40 min. Glucan conversion (wt %), total sugar yield (wt % pulp), and enzyme-treated solids recovered (wt % pulp) were calculated using Equations (1)–(3), respectively.

(1) $$\mathrm{Glucan}\;\mathrm{conversion}\;\left(\mathrm{wt}\;\%\right)\;=\frac{\mathrm{Glucose}\;\left(\mathrm{wt}\right)\times 100}{W_{1}},$$

where Glucose (wt) = mass of glucose calculated from HPLC analysis (g), *W*_1_ = mass of glucose in the pulp (determined from compositional analysis) used for enzyme hydrolysis (g)

(2) $$\mathrm{Total}\;\mathrm{mass}\;\mathrm{loss}\;\left(\mathrm{wt}\;\%\;\mathrm{pulp}\right)=\frac{\mathrm{Glucose}\;\left(\mathrm{wt}\right)\times \mathrm{AHC}\times 100}{W_{2}},$$

where AHC = the anhydro correction factor to convert mass of glucose to cellulose (0.9), *W*_2_ = mass of pulp used for enzyme hydrolysis (10 g)

Enzyme-treated solids recovered (wt % pulp) = 100 − Total mass loss (wt % pulp) (3)

### 2.5. Sample Preparation and Acid Hydrolysis

The undigested solids were centrifuged and washed three times with Milli-Q water at a centrifugation speed of 33,700× *g* for 10 min for each wash. The sugar-free solids were then freeze-dried and subjected to acid hydrolysis. The method used for bench scale acid hydrolysis was previously described by Beyene et al. [32] and was directly adopted from InnoTech Alberta’s Biomass Conversion and Processing Unit’s protocol (Edmonton, Alberta). The endoglucanase-treated solids (8 g, dry weight) were mixed with 100 mL of 64 wt % sulfuric acid (95–98%, Sigma-Aldrich, St. Louis, MO, USA) in 500 mL shake flasks to get a pulp to acid ratio of 8% (w/v). The flasks were placed in a water bath set at 45 °C, and were subjected to overhead stirring at 200 rpm. After a reaction time of 2 h, the reaction was quenched through addition of 10-fold (v/v) cold water. The suspension was then centrifuged for 10 min at 6400× *g* and some of the dilute acid was decanted. The pellet was resuspended in water to a final volume of 100–150 mL, and then neutralized with 4 M sodium hydroxide (98.8% Fisher Scientific; Hampton, NH, USA) to a final pH of 7.0. To help minimize the salt content, the suspension was centrifuged at 3700× *g* for 10 min, and the supernatant was decanted. The pellet was resuspended in water to a final volume of 20–70 mL, and placed into regenerated cellulose membrane tubing (Spectrum^TM^ Spectra/Por^TM^, Rancho Dominguez, CA, USA, 12–14 kDa molecular weight cut off) to facilitate dialysis. A conductivity meter was used to monitor the ionic strength of the liquid used for dialysis for 3–5 days. Once the conductivity was measured at 100–150 μS/cm, the suspension was centrifuged at 8900× *g* for 10 min, which facilitated precipitation of oversize particles. The colloidal CNC suspensions were collected, and the pellet was washed with water in order to lower the ionic strength and extract any remaining CNCs. An aliquot was taken from the pooled supernatants and oven-dried overnight at 103 °C. CNC recovery (wt % acid hydrolyzed feedstock) and CNC yield (wt % initial feedstock) were calculated using Equations (4) and (5), respectively, as shown below:

(4) $$\mathrm{CNC}\;\mathrm{recovery}\;\left(\mathrm{wt}\;\%\;\mathrm{acid}\;\mathrm{hydrolyzed}\;\mathrm{feedstock}\right)=\frac{W_{4}\times R}{W_{3}}\times \;100\;$$

where *W_3_* = mass of feedstock used in the acid hydrolysis (8 g), *W*_4_ = mass of oven-dried aliquot of CNCs (g), *R* = ratio of the aliquot to the total mass of the colloid

(5) $$\begin{array}{l} \mathrm{CNC}\;\mathrm{yield}\;\left(\mathrm{wt}\;\%\;\mathrm{initial}\;\mathrm{feedstock}\right)\\ =\frac{\mathrm{CNC}\;\mathrm{recovery}\;\left(\mathrm{wt}\;\%\;\mathrm{acid}\;\mathrm{hydrolyzed}\;\mathrm{feedstock}\right)\times \mathrm{Enzyme}–\mathrm{treated}\;\mathrm{solids}\;\mathrm{recovered}\;\left(\mathrm{wt}\;\%\;\mathrm{pulp}\right)\;}{100} \end{array}$$

### 2.6. Characterization

#### 2.6.1. X-ray Diffraction (XRD)

The crystalline structures of endoglucanase-treated wood pulp and CNCs were analyzed using a Rigaku Ultima IV X-ray diffractometer (Rigaku, Scottsdale, AZ, USA) with CuKα radiation (wavelength of 0.154 nm). The operating voltage and current were 40 kV and 44 mA, respectively. The samples were scanned at 2°/min with a 2θ angle ranging from 5° to 40°. The crystallinity index (CrI) value was calculated according to Segal et al. [35] and is shown in Equation (6) below.

(6) $$\;\mathrm{CrI}\;\left(\%\right)=\frac{I_{t}-I_{am}}{I_{t}}\times 100$$

where *I_t_* = the highest total crystalline peak at a diffraction angle around 2θ = 22.5°, *I_am_* = the amount of amorphous cellulose around at a diffraction angle around 2θ = 18°.

#### 2.6.2. Fourier Transform Infrared Spectroscopy (FTIR)

FTIR spectra of freeze-dried endoglucanase-treated pulp and CNCs were measured on a Thermo Nicolet iS50 FTIR spectrometer (Thermo Fisher Scientific, Madison, WI, USA) in attenuated total reflectance (ATR) mode and equipped with a diamond probe. The scan range was from 4000 to 400 cm^−1^, with a resolution of 4 cm^−1^. The spectra were averaged from 64 repeating scans per sample and the raw spectra were treated with auto baseline correction and advanced ATR correction. Three replicated measurements were recorded for each sample. The crystalline index (CI) was taken from the ratio of absorbance (%) from 1431 to 900 cm^−1^, which is attributed to the crystallinity index of cellulose I [36]. When FTIR experiments were performed on endoglucanase-treated samples, solid wood pulp fiber residues and CNCs obtained from 1.0 U/g of pulp treatment were used.

#### 2.6.3. Dynamic Light Scattering (DLS) and Zeta Potential Analyzer

CNC colloids were first diluted to 0.1 wt % in Milli-Q water. The 0.1 wt % dispersions were then diluted 1:1 with 10 mM NaCl to obtain a 0.05 wt % CNC dispersion in a 5 mM NaCl solution. The prepared CNC suspensions were filtered through a 0.45 µm syringe filter into sample cuvettes. The samples were analyzed on a Malvern Zetasizer Nano-ZS (Malvern Instruments Inc., Montreal, QC, Canada) for DLS and zeta potential analysis. The Zetasizer software (Zetasizer Software 7.11 provided by Malvern Instruments Inc., Montreal, QC, Canada) generates triplicate data from each experimental replicate (triplicate). 

#### 2.6.4. Transmission Electron Microscopy (TEM)

TEM images were captured using a Philips/FEI, Morgagni 268 TEM (Hillsboro, OR, USA) with 80 kV accelerating voltage. CNC colloids were diluted to 0.1 wt %, and a droplet was mounted on a formvar-coated 300-mesh copper grid (Ted Pella Inc., Redding, CA, USA). After 15 min, filter paper was used to carefully remove the excess liquid. Staining was achieved by exposing the specimen to phosphotungstic acid (2%, w/v) for 15 s. The excess stain was carefully removed using a filter paper before placing the sample onto the TEM sample holder.

#### 2.6.5. Differential Scanning Calorimetry (DSC)

Freeze-dried CNCs and endoglucanase-treated pulp samples were analyzed on a TA Q1000 DSC (TA Instruments, New Castle, DE, USA). Samples (4–8 mg) were weighted into aluminum pans. The temperature profile ranged from 30 °C to 350 °C with a heating rate of 10 °C/min. The nitrogen flow rate was set to 60 mL/min.

### 2.7. Statistical Analysis

Statistical analysis was done using SPSS Version 25 (IBM, Armonk, NY, USA). One-way ANOVA with Tukey’s HSD test was conducted for determining the significant level per experimental conditions with a confidence interval of 95%. All experiments were done in triplicates, unless stated otherwise.

## 3. Results and Discussion

### 3.1. Enzymatic Hydrolysis and Sugar Yield

**Figure 1:**
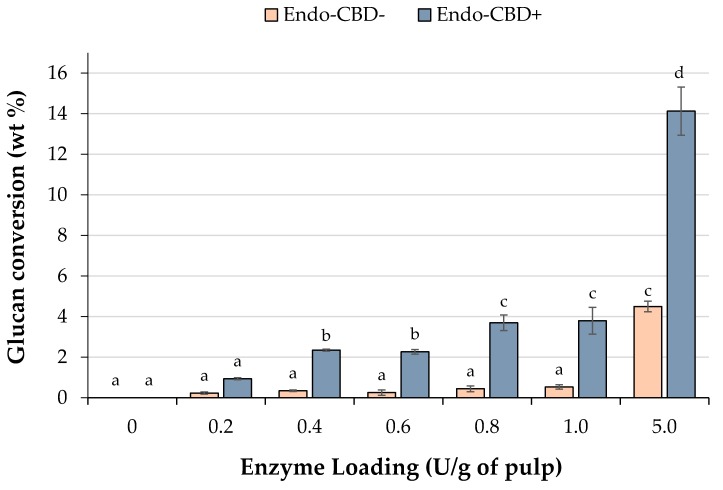
Glucan conversion from hydrolysis of wood pulp with endoglucanases. Wood pulp was subjected to enzyme hydrolysis at various loadings of endoglucanases with (Endo-CBD+) or without (Endo-CBD−) a cellulose-binding domain. Since endoglucanases generate long/short chain sugars rather than monosaccharides (i.e., glucose), quantification of the sugars released from wood pulp into the supernatant through endoglucanase action was achieved through a secondary enzymatic hydrolysis that enabled complete hydrolysis of sugars to glucose monomers. Columns labelled with different letters indicate means that are statistically different at a 95% confidence level.

Endoglucanases were used to selectively digest amorphous regions of wood pulp without damaging the crystalline cellulose domains. Endoglucanases with (Endo-CBD+) and without (Endo-CBD−) a cellulose-binding domain were selected for enzymatic hydrolysis. Since endoglucanases only cleave cellulose into fractions with different chain lengths, limited monosaccharides are found in the hydrolysates. Therefore, to evaluate the recoverable sugars released from wood pulp through endoglucanase activity, a second enzymatic hydrolysis was conducted using a cellulase cocktail with high activity. Glucose was the only sugar detected in the enzymatic hydrolysates, which indicates that the two endoglucanase cocktails lack activity on the xylan chains. Endo-CBD+ generally released more polysaccharide chains (that were subsequently hydrolyzed to glucose through a secondary enzyme hydrolysis) than Endo-CBD−, when the two endoglucanases were compared at constant enzyme loading (Figure 1). Karita et al. compared endoglucanase activity with and without a CBD, and reported that, in the latter case, the enzyme showed poor adsorption, and at low cellulose concentration, the enzymes exhibited low levels of binding [29].

The control group with no enzyme addition did not show any glucose release, which indicated that no free sugars were associated with the original wood pulp. Thus, any glucose released likely resulted from the addition of endoglucanase. Glucose yield from the Endo-CBD+ enzyme hydrolysis was quite low (less than 4%) until the 5.0 U/g of pulp enzyme loading was employed, where 14.1 ± 1.2 wt % glucan conversion was observed. Conversely, when the Endo-CBD− endoglucases was used, virtually no glucose (≤0.5 wt %) was released from the wood pulp, except for the 5.0 U/g of pulp loading where 4.5 ± 0.3 glucan conversion (wt %) was observed. This yield was statistically similar to the amount of glucose released using the 0.8 U/g of pulp and 1.0 U/g of pulp Endo-CBD+ loadings (Figure 1). It should be noted that the levels of glucose yield using the two endoglucanases were significantly lower than the extents (20–40%) observed by Beyene et al. from wood pulp hydrolysis using a highly processive cellulase cocktail. In addition, 6–12% xylose release was reported in these experiments, while the endoglucanases used in the present study were unable to hydrolyze hemicellulose [32]. Zhu et al. also observed that endoglucanase (Fibercare^®^) hydrolysis of softwood pulp was very poor, with nearly 100% solid yield recovered [37]. A cocktail enzyme composed of all three classes of cellulases (Celluclast 1.5 L), on the other hand, nearly resulted in 40% degradation. It was suggested that the endoglucanase activity was limited to chain cutting, while the cocktail led to complete hydrolysis of cellulose to monosaccharides. Nevertheless, it is possible that the action of endoglucanases can partially hydrolyze amorphous regions, making them more amenable to acid hydrolysis, and that future experiments can incorporate a small amount of cellobiohydrolase and/or xylanase.

### 3.2. CNC Yields from Acid Hydrolysis

**Figure 2:**
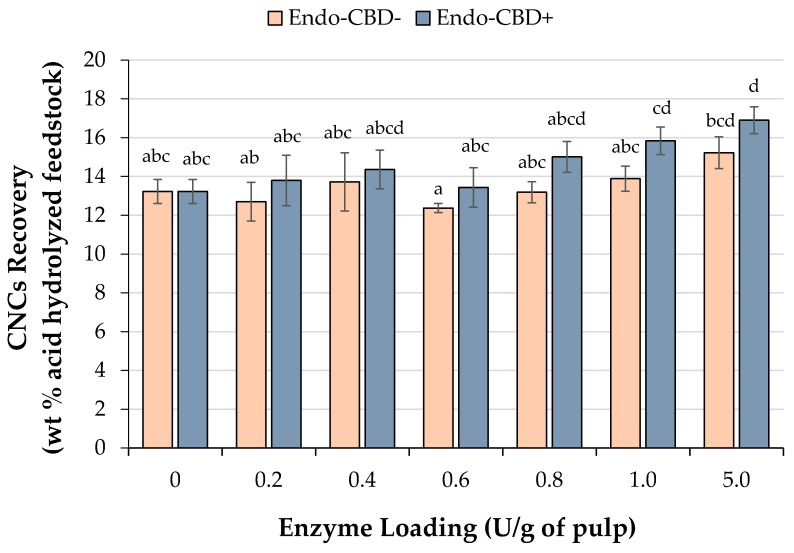
Cellulose nanocrystals (CNCs) recovered following acid hydrolysis of untreated wood pulp or the solid residue remaining after treatment with various amounts of endoglucanase with (Endo-CBD+) or without (Endo-CBD−) a cellulose-binding domain. CNC yields were determined by dividing the mass of CNCs obtained by the mass of the material subjected to acid hydrolysis (8 g). Columns labelled with different letters indicate means that are statistically different at a 95% confidence level.

The solid residues remaining after the endoglucanase treatments were then subjected to standard acid hydrolysis for production of CNCs. The CNC recovery (wt % acid hydrolyzed feedstock) was then calculated based on the aliquot of endoglucanase-treated wood pulp going into the acid hydrolysis reactor. Incorporation of the mock-treated wood pulp (no enzyme) resulted in a CNC recovery of 13.2 ± 0.6%. This increased to maxima of 16.9 ± 0.7% and 15.2 ± 0.8% when acid hydrolyses were performed using the solid residues obtained through Endo-CBD+ and Endo-CBD− treatment at 5.0 U/g of pulp loading, respectively (Figure 2). When Endo-CBD+ was used, a 1.0 U/g of pulp loading was the minimum enzyme loading that resulted in an increased CNC recovery following acid hydrolysis, relative to the no enzyme control. Conversely, for Endo-CDB-, a higher enzyme loading of 5.0 U/g of pulp was required before the CNC recovery obtained through acid hydrolysis was significantly higher than the mock-treated sample. It is worth noting that although increasing the enzyme loadings from 1.0 to 5.0 U/g of pulp resulted in a dramatic increase in the amount of recoverable sugar for both enzymes (Figure 1), there was no difference in the CNC recovery observed. With higher sugar yields, the endoglucanase treatment is expected to improve the concentration of crystalline cellulose as these enzymes have specific activity towards amorphous cellulose. However, it can be suggested that the extent of degradation obtained in the present study may not generate a significant concentration of crystals with the level of perfection/ordering of CNC precursor materials. Hence, the highly concentrated acid will likely dissolve these crystals as the lower level of ordering permits penetration and subsequent hydrolysis, resulting in a similar CNC recovery.

Overall, the improvements (1.2× and 1.3× from Endo-CBD− and Endo-CBD+ treatments, respectively) in CNC recovery from the acid hydrolysis process may have a notable impact in reducing the amount of acid and reagents, energy, and time requirements of subsequent CNC purification processes [32].

**Figure 3:**
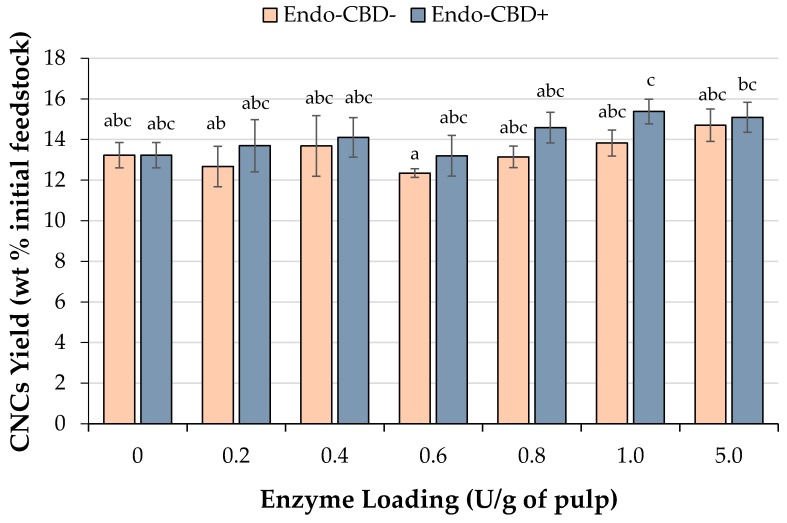
CNC yield of the overall endoglucanase enzymatically-mediated acid hydrolysis process. The yield from the initial feedstock was calculated by taking into account the mass loss due to endoglucanase enzymatic treatment. Columns labelled with different letters indicate means that are statistically different at a 95% confidence level.

The CNC yield (wt % initial feedstock) was calculated to evaluate the impact of mass loss conferred by enzymatic digestion on the yield. There was no significant change in the overall yield from both endoglucanase enzymatically-mediated processes, even with 15.3 ± 1.3 wt % glucan conversion, relative to the mock-treated wood pulp (Figure 3). Hence, cellulosic precursors for CNCs were not degraded by the enzymes, implying that the sugar release from the endoglucanases was from selective degradation of amorphous cellulose chains. The widely accepted understanding of cellulase activity is that endoglucanases attack amorphous cellulose, unlike cellobiohydrolases, which can degrade crystalline cellulose. It is postulated that endoglucanases have an open groove-like active site to allow binding of internal chains of amorphous regions and decrease the degree of polymerization of cellulose. On the other hand, cellobiohydrolases have a tunnel-shaped active site for processive catalysis of crystalline domains [26,38]. We have previously reported similar results on the overall CNC yields from acid hydrolysis of wood pulp treated with a cellulase cocktail composed of all three classes of enzymes for 2–8 h [32]. However, longer enzymatic treatment, for 10 h, reduced yields, suggesting that the presence of cellobiohydrolases is likely to confer damage to cellulosic precursors for CNCs.

In summary, these results show the potential of the Endo-CBD+ mediated acid hydrolysis process in recovering fermentable glucose and improving the CNC recovery process without compromising the yield. However, further investigations are required to improve the efficiency of endoglucanase hydrolysis to obtain sufficient sugar release for an economically feasible integrated ethanol co-production system. A feasibility study could shed light into how much sugar release and improvement in CNC recovery is required to incorporate an enzymatic treatment with meaningful impact on the process economics of acid hydrolysis.

### 3.3. Characterization of Endoglucanase-Treated Wood Pulp and Resulting CNCs

The CNCs generated through acid hydrolysis of the mock-treated or endoglucanase-treated wood pulp were then subjected to several analyses to assess their characteristics. In almost all cases, X-ray diffraction, dynamic light scattering, zeta potential, transmission electron microscopy, and differential scanning calorimetry analyses demonstrated that the CNCs generated from the solid residue remaining after treatment with endoglucanases (both Endo-CBD+ and Endo-CBD−) exhibited qualities that were similar to those generated from mock-treated wood pulp. A more detailed discussion of these results is found below.

#### 3.3.1. Crystallinity Index of Endoglucanase-Treated Wood Pulp

• X-ray diffraction (XRD) analysis

**Figure 4:**
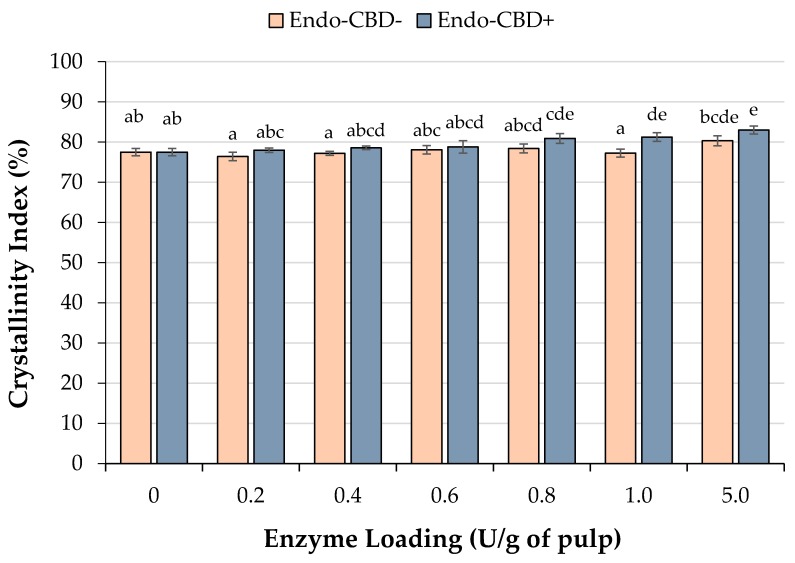
Crystallinity index as determined by XRD analysis. The solid residue recovered after treatment (or mock-treatment) of wood pulp with endoglucanases with (Endo-CBD+) or without (Endo-CBD−) cellulose-binding domains were subjected to XRD analysis to determine crystallinity. Columns labelled with different letters indicate means that are statistically different at a 95% confidence level.

To determine whether the endoglucanase treatment affected the crystallinity index of the wood pulp, XRD analysis was performed (Figure 4). In the case of Endo-CBD+ treatment, a significant increase in crystallinity index, from 77% up to 83%, was observed when the wood pulp was treated with ≥0.8 U/g of pulp enzyme loading. Conversely, for the treatment of wood pulp with Endo-CBD−, the crystallinity index of CNCs derived from all enzyme loadings were identical to the mock-treated control. XRD analysis of Eucalyptus holocellulose, hydrolyzed using a combination of endoglucanase and β-glucosidase, showed an increase in crystallinity index from 72% to 81% [22], which was similar to the results achieved using Endo-CBD+. The findings from our experiments provide further evidence that endoglucanases are able to degrade amorphous regions of cellulose in wood pulp to promote increased crystallinity and CNC yields. However, it should be noted that higher dosages of enzymes are desired to achieve both high oligosaccharide/sugar release and CNC yields.

• Fourier transformed infra-red (FTIR) analysis

In order to confirm the crystallinity index results obtained through XRD, we also employed a secondary approach based on ATR-FTIR to analyze fibers generated from enzymatic treatment with 1.0 U/g of pulp loading, and the resulting CNCs from acid hydrolysis. The cellulose I crystallinity index was calculated from the ratio of absorbances at 1431 to 900 cm^−1^, according to a method proposed by Kuo and Lee [39]. For this calculation, the absorption band at 1430 cm^−1^ is assigned to CH_2_ bending in crystalline cellulose; this band shifts to 1420 cm^−1^ in cellulose II and amorphous cellulose. Conversely, the absorption band at 900 cm^−1^, which has been assigned to C–O–C stretching of glycosidic bonds, is a marker of cellulose II and amorphous cellulose, as this band becomes weak and broad in cellulose I [36]. Based on the cellulose I crystallinity index as determined through FTIR, the solid fiber residue remaining after endoglucanase treatment displayed improved crystallinity index compared to the mock-treated wood pulp (Table 1). On the other hand, after acid hydrolysis, the crystallinity index of the CNCs generated from mock-treated (1.6 ± 0.1) or Endo-CBD+-treated (1.0 U/g of pulp) wood-pulp (1.4 ± 0.1) was statistically similar. Interestingly, while XRD analysis indicated that the Endo-CBD− treatment had no effect on crystallinity index of CNCs, ATR-FTIR suggested that the Endo-CBD− treatment actually resulted in a lower crystallinity index. These results highlight the need for a more reliable method of crystallinity index determination. Khatri et al. recently described the use of fluorescent markers to quantify the amount of crystalline and amorphous celluloses, which may serve as a better method for determination of crystallinity index [40].

#### 3.3.2. Dynamic Light Scattering (DLS) and Zeta Potential (ZP) Analysis of CNCs Suspensions

Additional analyses were then performed to characterize the suspensions of CNCs produced following acid hydrolysis. First, particle sizes and size distributions were assessed using dynamic light scattering analysis (DLS). For these experiments, CNCs generated through acid hydrolysis of the solid residue derived from treatment of wood pulp with endoglucanases (1.0 U/g of pulp loading) were compared to the mock-treated control. The average hydrodynamic diameter of CNCs from mock-treated wood pulp was 144.6 ± 2.3 nm, with 92.9 ± 5.5% particles in the same distribution (Table 2). The average diameter of CNCs isolated from wood pulp treated with Endo-CBD− (147.7 ± 7.8 nm) was comparable to the mock-treated samples. This was not observed for CNCs obtained through acid hydrolysis of solid residue generated from Endo-CBD+ treatment, which displayed a smaller average diameter of 125.2 ± 7.4 nm. Bondeson et al. obtained CNCs with an average diameter of 200 to 400 nm from acid hydrolysis of microcrystalline cellulose derived from Norway Spruce [41]. The average hydrodynamic diameter in the present study seems relatively narrow, most likely because the colloid was passed through a 0.45 µm filter.

Zeta potential measurement evaluates the density and nature of surface charges. Higher values of zeta potential indicate a higher capacity of dispersion in water, while lower values indicate low dispersion stability. Based on the literature, an absolute value greater than 40 mV reflects good stability of a colloidal suspension [42]. As shown in Table 2, CNCs obtained from acid hydrolysis of endoglucanase-treated wood pulp had zeta potential values ≤ −40 mV, which confirms their colloidal stability. The stability of CNCs dispersions is due to the electrostatic repulsion between the negative sulphate groups (–OSO_3_^−^) on the surface of the cellulose [43], which are derived from esterification of the hydroxyl groups present on the cellulose surface. Vasconcelos et al. [42] reported that higher H_2_SO_4_ concentrations and prolonged reaction times increased the absolute zeta potential value, favoring the stability of such CNC suspensions.

#### 3.3.3. Transmission Electron Microscopy (TEM) Images of CNCs

**Figure 5:**
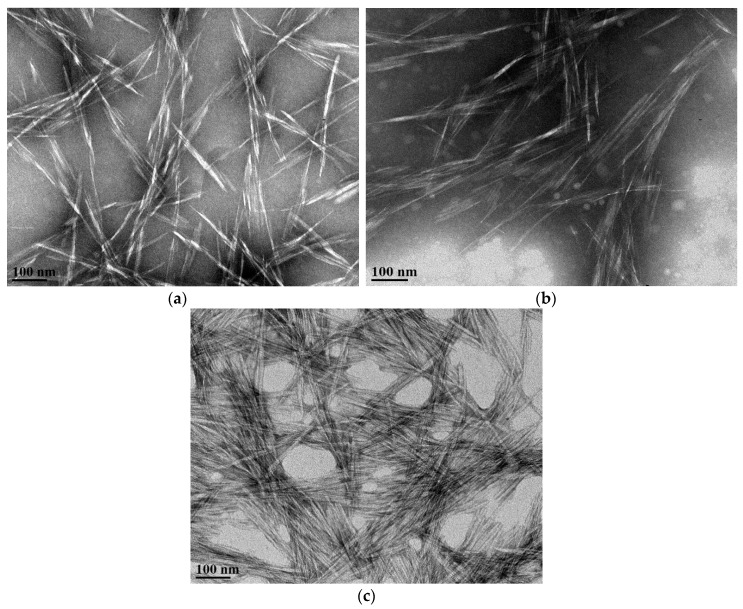
TEM images of cellulose nanocrystals produced from acid hydrolysis of (**a**) mock-treated wood pulp (**b**) Endo-CBD+ (1.0 U/g of pulp loading)-treated wood pulp, and (**c**) Endo-CBD− (1.0 U/g of pulp loading)-treated wood pulp.

TEM micrographs of CNCs derived from acid hydrolyzed mock-treated and endoglucanase-treated (Endo-CBD+ or Endo-CBD−) wood pulp are shown in Figure 5. The CNC particles generated from the three different acid hydrolysis feedstocks appear similar in size, with all particles adopting an expected needle shape form, as reported in other studies [5,42].

#### 3.3.4. Differential Scanning Calorimetry (DSC)

**Figure 6:**
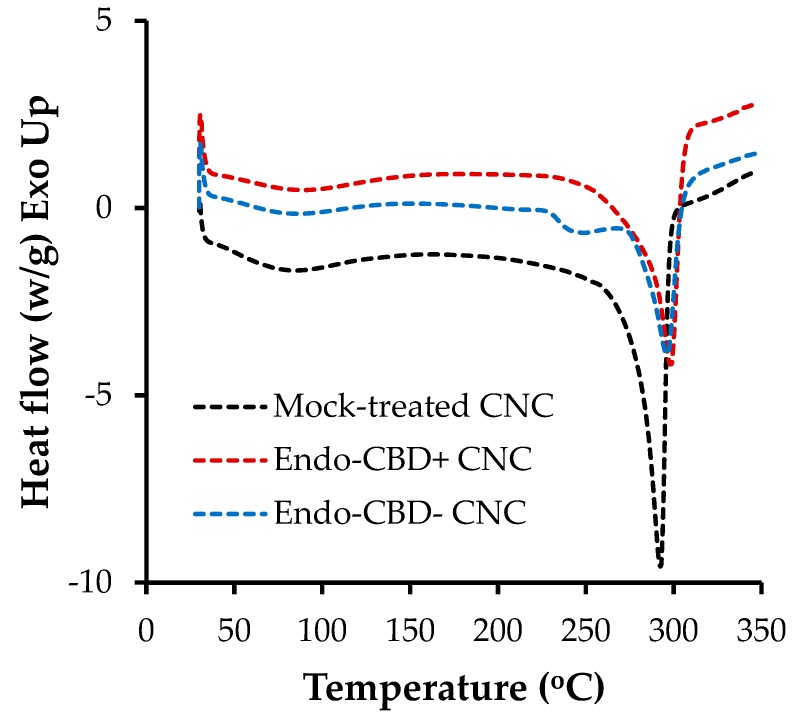
DSC thermograms of CNC samples. The CNCs that were generated through acid hydrolysis of wood pulp subjected to a mock-treatment or a treatment with endoglucanases with (Endo-CDB+) or without (Endo-CBD−) a cellulose-binding domain (1.0 U/g of pulp loading) were analyzed via DSC. The results shown above represent the average of duplicate experiments.

The heat transition curves generated from DSC study of the CNCs samples are shown in Figure 6. All the curves showed an initial drop between 50 °C and 150 °C, which was likely due to moisture loss. For all three CNCs samples, degradation commenced at roughly 290 °C to 300 °C, as evidenced by the large thermal event observed in the DSC thermograms at this temperature range. Similar DSC profiles were also reported in other studies in the literature [42,44,45]. There was no difference in the thermal transition temperatures between the CNCs generated from the mock and enzyme-treated wood pulp. This further confirms that the endoglucanase treatment in the present study did not result in changes to the CNCs generated through subsequent acid hydrolysis.

## 4. Conclusions

Enzymatic hydrolysis of wood pulp using endoglucanases has shown potential to improve the yields of CNCs and produce a fermentable monosaccharide by-product stream. In general, the use of endoglucanases with a cellulose-binding domain (Endo-CBD+) produced higher sugar yields and improved crystallinity of wood pulp when compared to the use of endoglucanases without a cellulose-binding domain (Endo-CBD−). The CNCs produced from the solid residue remaining after endoglucanase treatment showed similar characteristics compared to CNCs produced from traditional acid hydrolysis. However, given the relatively low amounts of sugar released from endoglucanase treatment, future research will examine a combination of enzymes, such as endoglucanase, xylanase, and β-glucosidase, to remove both the hemicellulose and completely hydrolyze the amorphous cellulose in the wood pulp to monosaccharides prior to acid hydrolysis. An advanced enzyme system such as this would be expected to improve both the CNC and monosaccharide yield. Nevertheless, this study serves as an important benchmark for future studies, demonstrating that endoglucanase treatment of wood pulp does not negatively impact the quality of CNCs produced from subsequent acid hydrolysis.

## Figures and Tables

**Table 1:** FTIR assessment of crystallinity index of mock-treated or endoglucanase-treated (1.0 U/g of pulp) wood pulp and resulting CNCs generated through acid hydrolysis.

Samples	Crystalline Index (Absorbance Ratio: 1430 cm^−1^/900 cm^−1^)
Pulp:	
Mock-treated	0.7 ± 0.0^A^
Endo-CBD+-treated	1.1 ± 0.1^B^
Endo-CBD−-treated	1.0 ± 0.1^B^
CNCs:	
Mock-treated	1.6 ± 0.1^a^
Endo-CBD+-treated	1.4 ± 0.1^ab^
Endo-CBD−-treated	1.3 ± 0.1^b^

Note: Within the two groups, treatments annotated with different letters (i.e., A vs. B, or a vs. b) are statistically different, at a 95% confidence level.

**Table 2:** Dynamic light scattering (DLS) analysis for particles size and zeta potential (ZP) measurements of CNCs extracted from untreated and endoglucanase-treated (1.0 U/g of pulp loading) wood pulp.

CNC Sample	Average Hydrodynamic Diameter (nm)	Intensity (%)	Polydispersity Index *	Zeta Potential (mV)
Mock-treated	144.6 ± 2.3	92.9 ± 5.5	0.3 ± 0.1	−40.1 ± 1.8
Endo-CBD+-treated	125.2 ± 7.4	92.5 ± 6.7	0.3 ± 0.0	−43.6 ± 1.0
Endo-CBD−-treated	147.7 ± 7.8	93.7 ± 2.9	0.3 ± 0.0	−40.3 ± 0.2

* values < 0.7 are considered homogenous.

## References

[1] Resolute Forest Products Building the Resolute of the Future: 2015 Annual Report. http://www.resolutefp.com/uploadedFiles/Media/Publications/Resolute_2015_Annual_Report.pdf.

[2] Natural Resources Canada Nanotechnology Applications in the Forest Sector. http://www.cfs.nrcan.gc.ca/pubwarehouse/pdfs/29382.pdf.

[3] Brinchi L., Cotana F., Fortunati E., Kenny J.M. (2013). Production of nanocrystalline cellulose from lignocellulosic biomass: Technology and applications. Carbohydr. Polym..

[4] Wang B., Sain M., Oksman K. (2007). Study of Structural Morphology of Hemp Fiber from the Micro to the Nanoscale. Appl. Compos. Mater..

[5] Lamaming J., Hashim R., Sulaiman O., Leh C.P., Sugimoto T., Nordin N.A. (2015). Cellulose nanocrystals isolated from oil palm trunk. Carbohydr. Polym..

[6] Kümmerer K., Menz J., Schubert T., Thielemans W. (2011). Biodegradability of organic nanoparticles in the aqueous environment. Chemosphere.

[7] Moon R.J., Martini A., Nairn J., Simonsen J., Youngblood J. (2011). Cellulose nanomaterials review: Structure, properties and nanocomposites. Chem. Soc. Rev..

[8] Dufresne A. (2008). Polysaccharide nanocrystal reinforced nanocomposites. Can. J. Chem..

[9] Habibi Y., Lucia L.A., Rojas O.J. (2010). Cellulose Nanocrystals: Chemistry, Self-Assembly, and Applications. Chem. Rev..

[10] Siqueira G., Bras J., Dufresne A. (2010). Cellulosic Bionanocomposites: A Review of Preparation, Properties and Applications. Polymers.

[11] Visakh P., Thomas S. (2010). Preparation of Bionanomaterials and their Polymer Nanocomposites from Waste and Biomass. Waste Biomass Valoriz..

[12] Abdul Khalil H.P.S., Bhat A.H., Ireana Yusra A.F. (2012). Green composites from sustainable cellulose nanofibrils: A review. Carbohydr. Polym..

[13] Bettaieb F., Khiari R., Hassan M.L., Belgacem M.N., Bras J., Dufresne A., Mhenni M.F. (2015). Preparation and characterization of new cellulose nanocrystals from marine biomass Posidonia oceanica. Ind. Crops Prod..

[14] Peng B.L., Dhar N., Liu H.L., Tam K.C. (2011). Chemistry and applications of nanocrystalline cellulose and its derivatives: A nanotechnology perspective. Can. J. Chem. Eng..

[15] Teixeira E.D.M., Bondancia T.J., Teodoro K.B.R., Corrêa A.C., Marconcini J.M., Mattoso L.H.C. (2011). Sugarcane bagasse whiskers: Extraction and characterizations. Ind. Crops Prod..

[16] Kilpeläinen I., Xie H., King A., Granstrom M., Heikkinen S., Argyropoulos D.S. (2007). Dissolution of wood in ionic liquids. J. Agric. Food Chem..

[17] Qin Z.Y., Tong G.L., Chin Y.C.F., Zhou J.C. (2011). Ultrasonic assisted synthesis of two new coordination polymers and their applications as precursors for preparation of nano-materials. BioResources.

[18] Siró I., Plackett D. (2010). Microfibrillated cellulose and new nanocomposite materials: A review. Cellulose.

[19] Welton T. (1999). Room-temperature ionic liquids. Solvents for synthesis and catalysis. Chem. Rev..

[20] Duran N., Lemes A.P., Duran M., Freer J., Baeza J. (2011). A minireview of cellulose nanocrystals and its potential integration as co-product in bioethanol production. J. Chil. Chem. Soc..

[21] Henriksson G., Henriksson M., Berglund L.A., Lindström T. (2007). An environmentally friendly method for enzyme-assisted preparation of microfibrillated cellulose (MFC) nanofibers. Eur. Polym. J..

[22] Teixeira R.S.S., Silva A.S.A.D., Jang J.H., Kim H.W., Ishikawa K., Endo T., Lee S.H., Bon E.P.S. (2015). Combining biomass wet disk milling and endoglucanase/β-glucosidase hydrolysis for the production of cellulose nanocrystals. Carbohydr. Polym..

[23] Xu Y., Salmi J., Kloser E., Perrin F., Grosse S., Denault J., Lau P.C.K. (2013). Feasibility of nanocrystalline cellulose production by endoglucanase treatment of natural bast fibers. Ind. Crops Prod..

[24] Béguin P., Aubert J.P. (1994). The biological degradation of cellulose. FEMS Microbiol. Rev..

[25] Gan Q., Allen S.J., Taylor G. (2003). Kinetic dynamics in heterogeneous enzymatic hydrolysis of cellulose: An overview, an experimental study and mathematical modelling. Process Biochem..

[26] Zhang Y.H.P., Lynd L.R. (2004). Toward an aggregated understanding of enzymatic hydrolysis of cellulose: Noncomplexed cellulase systems. Biotechnol. Bioeng..

[27] Lynd L.R., Weimer P.J., van Zyl W.H., Pretorius I.S. (2002). Microbial cellulose utilization: Fundamentals and biotechnology. Microbiol. Mol. Biol. Rev..

[28] Boraston A.B., Bolam D.N., Gilbert H.J., Davies G.J. (2004). Carbohydrate-binding modules: Fine-tuning polysaccharide recognition. Biochem. J..

[29] Karita S., Sakka K., Ohmiya K. (1996). Cellulose-binding domains confer an enhanced activity against insoluble cellulose to Ruminococcus albus endoglucanase IV. J. Ferment. Bioeng..

[30] Shoseyov O., Shani Z., Levy I. (2006). Carbohydrate Binding Modules: Biochemical Properties and Novel Applications. Microbiol. Mol. Biol. Rev..

[31] Sandgren M., Shaw A., Ropp T.H., Wu S., Bott R., Cameron A.D., Stahlberg J., Mitchinson C., Jones T.A. (2001). The X-ray crystal structure of the Trichoderma reesei family 12 endoglucanase 3, Cel12A, at 1.9 A resolution. J. Mol. Biol..

[32] Beyene D., Chae M., Dai J., Danumah C., Tosto F., Demesa A.G., Bressler D.C. (2017). Enzymatically-Mediated Co-Production of Cellulose Nanocrystals and Fermentable Sugars. Catalysts.

[33] Jain T., Van Gerpen J., McDonald A. (2010). Production of Fuel Ethanol from Woody Biomass. J. Biofuels.

[34] Ghose T.K. (1987). Measurement of Cellulase Activites. Pure Appl. Chem..

[35] Segal L., Creely J.J., Martin J.A.E., Conrad C.M. (1959). An Empirical Method for Estimating the Degree of Crystallinity of Native Cellulose Using the X-Ray Diffractometer. Text. Res. J..

[36] Song Y., Zhang J., Zhang X., Tan T. (2015). The correlation between cellulose allomorphs (I and II) and conversion after removal of hemicellulose and lignin of lignocellulose. Bioresour. Technol..

[37] Zhu J., Sabo R., Clemons C. (2014). Methods for Integrated Conversion of Lignocellulosic Material to Sugars or Biofuels and Nano-Cellulose. U.S. Patent.

[38] Rouvinen J., Bergfors T., Teeri T., Knowles J.K., Jones T.A. (1990). Three-dimensional structure of cellobiohydrolase II from Trichoderma reesei. Science.

[39] Kuo C.H., Lee C.K. (2009). Enhancement of enzymatic saccharification of cellulose by cellulose dissolution pretreatments. Carbohydr. Polym..

[40] Khatri V., Meddeb-Mouelhi F., Adjallé K., Barnabé S., Beauregard M. (2018). Determination of optimal biomass pretreatment strategies for biofuel production: Investigation of relationships between surface-exposed polysaccharides and their enzymatic conversion using carbohydrate-binding modules. Biotechnol. Biofuels.

[41] Bondeson D., Mathew A., Oksman K. (2006). Optimization of the isolation of nanocrystals from microcrystalline cellulose by acid hydrolysis. Cellulose.

[42] Vasconcelos N.F., Feitosa J.P.A., da Gama F.M.P., Morais J.P.S., Andrade F.K., de Souza Filho M.D.S.M., Rosa M.D.F. (2017). Bacterial cellulose nanocrystals produced under different hydrolysis conditions: Properties and morphological features. Carbohydr. Polym..

[43] Tonoli G.H.D., Teixeira E.M., Corrêa A.C., Marconcini J.M., Caixeta L.A., Pereira-da-Silva M.A., Mattoso L.H.C. (2012). Cellulose micro/nanofibres from Eucalyptus kraft pulp: Preparation and properties. Carbohydr. Polym..

[44] Maiti S., Jayaramudu J., Das K., Reddy S.M., Sadiku R., Ray S.S., Liu D. (2013). Preparation and characterization of nano-cellulose with new shape from different precursor. Carbohydr. Polym..

[45] Mandal A., Chakrabarty D. (2011). Isolation of nanocellulose from waste sugarcane bagasse (SCB) and its characterization. Carbohydr. Polym..

